# Supplementary material for: “They recognize me as a doctor”: A peer mobilisation training programme to promote oral HIV self-testing and referral for acute HIV infection screening among gay and bisexual men and transgender women in coastal Kenya, an exploratory study
Source: PLoS One. 2025 Dec 4;20(12):e0322255. doi: 10.1371/journal.pone.0322255 (PMC12677470; doi:10.1371/journal.pone.0322255)
Supplement: S1 Table — (PDF) [file pone.0322255.s001.pdf]

S1 Table. Demographic characteristics of 18 Focus group discussion participants.

		Frequency (%)
**Gender**	Male	9 (50.0)
	Transgender woman	9 (50.0)
**Sexuality**	Gay	4 (22.2)
	Bisexual	9 (50.0)
	Other	5 (27.8)
**Religion**	Christian	10 (55.6)
	Muslim	6 (33.3)
	Other	2 (11.1)
**Highest level of education completed**	Primary	6 (33.3)
	Secondary	7 (38.9)
	Tertiary	4 (22.2)
	Prefer not to say	1 (5.6)
**Received payment for sex in the last six months**	Yes	18 (100.0)
**Experience as peer mobiliser**	Yes	18 (100)
**Experience mobilising with OSTs**	Yes	18 (100)
		**Median (IQR)**
**Age (years)**		26.5 (24-30)
**Median number of years of experience as peer educator/mobiliser**		4.5 (2-10)
