# Supplementary material for: “They recognize me as a doctor”: A peer mobilisation training programme to promote oral HIV self-testing and referral for acute HIV infection screening among gay and bisexual men and transgender women in coastal Kenya, an exploratory study
Source: PLoS One. 2025 Dec 4;20(12):e0322255. doi: 10.1371/journal.pone.0322255 (PMC12677470; doi:10.1371/journal.pone.0322255)
Supplement: S2 Table — (PDF) [file pone.0322255.s002.pdf]

S2 Table. Demographic characteristics of 15 In-Depth Interview participants.

		**Frequency (%)**
**Gender**	Male	15 (100.0)
	Transgender woman	0 (0.0)
**Sexuality**	Gay	0 (0.0)
	Bisexual	15 (100.0)
	Other	0 (0.0)
		**Median (IQR)**
**Age (years)**		25.5 (22-29)
