# Supplementary material for: “They recognize me as a doctor”: A peer mobilisation training programme to promote oral HIV self-testing and referral for acute HIV infection screening among gay and bisexual men and transgender women in coastal Kenya, an exploratory study
Source: PLoS One. 2025 Dec 4;20(12):e0322255. doi: 10.1371/journal.pone.0322255 (PMC12677470; doi:10.1371/journal.pone.0322255)
Supplement: S3 Table — (PDF) [file pone.0322255.s003.pdf]

S3 Table. Eligibility criteria of mobilised participants and index participants.

**Study population**	**Eligibility criteria**
Mobilised participants	Inclusion criteria:<br>• Age 18 years or older;<br>• Male sex assigned at birth;<br>• Reporting oral or anal sex with men in the previous six months;<br>• HIV negative or of unknown HIV status before doing the self-test;<br>• Willing to provide a blood sample;<br>• Reporting at least one behavioural factor or at least two AHI symptoms or at least one STI symptom[a]<br>o Behavioural factors: in the previous seven days: any condomless sex; or in the previous three months: sex with only men, receptive anal sex or group sex;<br>o AHI symptoms: in the previous 14 days: fever, diarrhoea, fatigue, body aches, sore throat;<br>o STI symptoms: in the previous 14 days: genital ulcer, genital discharge or anal discharge<br>Exclusion criteria:<br>• Well-adherent to PrEP
Index participants	Inclusion criteria:<br>• Age 18 years or older;<br>• Male sex assigned at birth;<br>• Reporting oral or anal sex with men in the previous six<br>• months;<br>• Being newly diagnosed with HIV, either after peer<br>• mobilisation, AHI screening, HPN or at the HTC of one<br>• of the study clinics during the study period (April through<br>• August 2019);<br>• Willing to provide a blood sample

AHI, acute HIV infection; PrEP, pre-exposure prophylaxis; STI, sexually transmitted infection. a. Based on published behavioural PrEP eligibility score (Wahome E et al. An Empiric Risk Score to Guide PrEP Targeting Among MSM in Coastal Kenya. AIDS Behav. 2018;22(Suppl 1):35- 44.) and AEHI symptom score (Sanders EJ et al. Targeted screening of at-risk adults for acute HIV-1 infection in sub-Saharan Africa. AIDS. 2015;29 Suppl 3:S221-30.)
