# Supplementary material for: “They recognize me as a doctor”: A peer mobilisation training programme to promote oral HIV self-testing and referral for acute HIV infection screening among gay and bisexual men and transgender women in coastal Kenya, an exploratory study
Source: PLoS One. 2025 Dec 4;20(12):e0322255. doi: 10.1371/journal.pone.0322255 (PMC12677470; doi:10.1371/journal.pone.0322255)
Supplement: S2 File — (PDF) [file pone.0322255.s005.pdf]

S2 File. Coding manual. FGDs with Peer Mobilisers.

Code		Sub-code	
**1**	Positive experiences	1.1	Improved skills
		1.2	Improved social network
**2**	Mobilisation process	2.1	Network access
		2.2	Knowledge sharing
**3**	Challenges of mobilisation	3.1	Coordinating with/persuading peers
		3.2	Logistics
		3.3	Stigma/security concerns
		3.4	Understanding
		3.5	Financial constraints
**4**	Overcoming challenges	4.1	Patience/perseverance
		4.2	Empower/support clients (procedure/benefits)
		4.3	Provide transport money
		4.4	Adjust mobilisation role
		4.5	Discretion
**5**	Resources	5.1	Opinions on resources
		5.2	Use of resources
**6**	OST	6.1	Knowledge of OST
		6.2	Use of OST
**7**	Confirmation test	7.1	Reluctance to return
		7.2	Do not want to know status
**8**	Ways to motivate GBT to test	8.1	Peer education event
		8.2	Encourage peers to test regularly
		8.3	Additional OSTs
**9**	AHI	9.1	Knowledge of AHI (understanding/sharing)
		9.2	Encountered client with symptoms
**10**	Challenges of AHI mobilisation	10.1	Timing of symptoms
		10.2	No target or financial incentive
		10.3	Peers sought treatment elsewhere
**11**	Experience of training	11.1	Positive aspects
		11.2	Negative aspects
		11.3	Feedback/discussions
		11.4	Practice mobilisation techniques
**12**		12.1	Continued involvement of mobilisers

	How to improve training/mobilisation	12.2	More training & resources
