# Supplementary material for: “They recognize me as a doctor”: A peer mobilisation training programme to promote oral HIV self-testing and referral for acute HIV infection screening among gay and bisexual men and transgender women in coastal Kenya, an exploratory study
Source: PLoS One. 2025 Dec 4;20(12):e0322255. doi: 10.1371/journal.pone.0322255 (PMC12677470; doi:10.1371/journal.pone.0322255)
Supplement: S3 File — (PDF) [file pone.0322255.s006.pdf]

# **S3 File. Coding manual.** Interviews with Index Clients.

| Code | | | Sub-code |
|---|---|---|---|
| **1** | Route of mobilisation | 1.1 | Hot spot/group |
| | | 1.2 | LGBTI+ organisation |
| | | 1.3 | Phone call/social media (*including WhatsApp*) |
| | | 1.4 | Peer educator/mobiliser |
| | | 1.5 | Referral by partner/acquaintance |
| | | 1.6 | Unclear |
| **2** | Information received | 2.1 | Risk behaviours |
| | | 2.2 | AHI |
| | | 2.3 | OST |
| | | 2.4 | PrEP |
| | | 2.5 | PEP |
| | | 2.6 | Clinic/testing centre |
| | | 2.7 | Recruitment procedures (*i.e. expenses, testing procedures etc.*) |
| | | 2.8 | Materials (*e.g. booklet, leaflet etc.*) |
| | | 2.9 | General testing procedures |
| | | 2.10 | General sexual health |
| | | 2.11 | ART |
| | | 2.12 | RNA testing |
| **3** | Information not received (*code if negative response to question*) | 3.1 | Risk behaviours |
| | | 3.2 | AHI |
| | | 3.3 | OST |
| | | 3.4 | PrEP |
| | | 3.5 | PEP |
| | | 3.6 | Clinic/testing centre |
| | | 3.7 | Recruitment procedures (*i.e. expenses, testing procedures etc.*) |
| | | 3.8 | Materials (*e.g. booklet, leaflet etc.*) |
| | | 3.9 | General testing procedures |
| | | 3.10 | General sexual health |
| **4** | Positive aspects of mobilisation | 4.1 | Patient/persistent |
| | | 4.2 | Motivating/supportive |
| | | 4.3 | Information/understandable |
| | | 4.4 | Confidential |
| | | 4.5 | Convenience |
| | | 4.6 | Beneficial for one's health |
| **5** | | 5.1 | Transport costs |

| | | | |
|---|---|---|---|
| | Support received from PM | 5.2 | Accompanied to clinic |
| | | 5.3 | Partner notification (*preferred approach or already conducted*) |
| | | 5.4 | Coping with diagnosis |
| | | 5.5 | Symptom referral |
| | | 5.6 | Understanding of test results |
| | | 5.7 | Keeping ART safe |
| **6** | Challenges of mobilisation | 6.1 | Understanding |
| | | 6.3 | Financial constraints |
| | | 6.3 | Stigma/security concerns |
| | | 6.4 | Logistical (*e.g. work commitments, Ramadan, weather*) |
| | | 6.5 | Risk of breach of confidentiality |
| **7** | Recommendations | 7.1 | Follow-up with index clients |
| | | 7.2 | Liaise with index clients |
| | | 7.3 | Use peer mobilisers for PNS |
| | | 7.4 | Organise educational meetings for MSM |
| | | 7.5 | RNA testing |
| | | 7.6 | Peer referral, CBOs, (targeted) outreaches |
| | | 7.7 | Social media |
| | | 7.8 | Give out health advice |
| | | 7.9 | Visit schools |
| | | 7.10 | Door-to-door outreach |
| | | 7.11 | Rapport building |
| **8** | Additional points | 8.1 | Shock |
| | | 8.2 | No prior information |
| **9** | Motivation for testing | 9.1 | Symptoms |
| | | 9.2 | Other |
