# Supplementary material for: “They recognize me as a doctor”: A peer mobilisation training programme to promote oral HIV self-testing and referral for acute HIV infection screening among gay and bisexual men and transgender women in coastal Kenya, an exploratory study
Source: PLoS One. 2025 Dec 4;20(12):e0322255. doi: 10.1371/journal.pone.0322255 (PMC12677470; doi:10.1371/journal.pone.0322255)
Supplement: Attachment — Submitted filename: HIV and AHI Education Pilot Study_Rebuttal Letter_01JUL2025.docx [file pone.0322255.s008.docx]

# Response to Reviewers

To the Academic Editor and Reviewers,

Thank you for taking the time to carefully review our manuscript on ““They recognize me as a doctor”: evaluation of an HIV peer mobilisation training programme to promote oral HIV self-testing and referral for acute HIV infection screening among gay and bisexual men and transgender women in coastal Kenya”. We are grateful for your insightful comments. We have addressed all of the requirements and comments detailed in the PLOS ONE Decision letter and provide a response to each comment in turn in this rebuttal letter. To note, any line mention refers to the line number in the marked-up copy of the manuscript to facilitate tracking of the changes relative to the submitted manuscript.

Sincerely,

On behalf of the co-authors,

Shaun Palmer

**Journal requirements**

1. Please ensure that your manuscript meets PLOS ONE's style requirements, including those for file naming.:
   - All style requirements have been applied
2. Please include a complete copy of PLOS’ questionnaire on inclusivity in global research in your revised manuscript. Our policy for research in this area aims to improve transparency in the reporting of research performed outside of researchers’ own country or community. The policy applies to researchers who have travelled to a different country to conduct research, research with Indigenous populations or their lands, and research on cultural artefacts. The questionnaire can also be requested at the journal’s discretion for any other submissions, even if these conditions are not met. Please find more information on the policy and a link to download a blank copy of the questionnaire here: https://journals.plos.org/plosone/s/best-practices-in-research-reporting. Please upload a completed version of your questionnaire as Supporting Information when you resubmit your manuscript.
   - The questionnaire has been completed and uploaded as a Supporting Information file. We have also provided extra information in the manuscript from lines **122-124** on how we facilitated inclusivity in global research by conducting focus group discussion with members and leaders of two LGBTI community organisations in coastal Kenya, during which they provided input to the study protocol.
   - From lines **168-171**, we also elaborated on how the training programme and communication materials were designed collaboratively with LGBTI study staff, representatives from two local LGBTI organisations, and mobilisers participating in the programme. This includes justification on lines **167-168** that we took this approach to develop the training programme and communication materials in a “*collaborative and inclusive way to ensure that all resources were culturally relevant and sensitive*.”
   - We further add from lines **174-176** detail on what aspects of the content that feedback was provided, stating that “*Partners were invited to share feedback and suggestions on all aspects of the resources, including comprehension of the overall content, the depth and breadth of the content, the quality of the English to Kiswahili translations, and the choice of imagery (i.e., graphics and icons)*.”
3. In the online submission form, you indicated that [All relevant data are within the manuscript and its Supporting Information files. Consisting of data provided by the authors to benefit the reader, the posted materials are not copyedited and are the sole responsibility of the authors, so questions or comments should be addressed to the corresponding author.].

   All PLOS journals now require all data underlying the findings described in their manuscript to be freely available to other researchers, either 1. In a public repository, 2. Within the manuscript itself, or 3. Uploaded as supplementary information.

   This policy applies to all data except where public deposition would breach compliance with the protocol approved by your research ethics board. If your data cannot be made publicly available for ethical or legal reasons (e.g., public availability would compromise patient privacy), please explain your reasons on resubmission and your exemption request will be escalated for approval.
   - All relevant data has been uploaded as Supporting Information in the file ‘Supplementary Materials_HIV AHI Peer Mobilisation Pilot Study_16JUN2025’. The supporting information captions are listed at the end of the revised manuscript (lines **722-728**)
4. When completing the data availability statement of the submission form, you indicated that you will make your data available on acceptance. We strongly recommend all authors decide on a data sharing plan before acceptance, as the process can be lengthy and hold up publication timelines. Please note that, though access restrictions are acceptable now, your entire data will need to be made freely accessible if your manuscript is accepted for publication. This policy applies to all data except where public deposition would breach compliance with the protocol approved by your research ethics board. If you are unable to adhere to our open data policy, please kindly revise your statement to explain your reasoning and we will seek the editor's input on an exemption. Please be assured that, once you have provided your new statement, the assessment of your exemption will not hold up the peer review process.
   - As per the response to comment 3, above, on supporting information, all necessary data is available in the Supplementary Materials, this includes an expanded list of additional quotes. We have also uploaded a full set of anonymized transcripts for the key informant interviews and focus group discussions, removing any identifiable information (such as age, dates, sexuality, and specific location) to respect the privacy of all participants.
5. Your ethics statement should only appear in the Methods section of your manuscript. If your ethics statement is written in any section besides the Methods, please move it to the Methods section and delete it from any other section. Please ensure that your ethics statement is included in your manuscript, as the ethics statement entered into the online submission form will not be published alongside your manuscript.
   - I can confirm that the ethics statement is only included in the Methods section.

**Editor comments**

1. *There are two main concerns with the paper.*
   a. *The first one is that it is not clear what type of evaluation authors are reporting. Is this about design, processes, impact? This is not clear neither in the objective nor in the methods. The described study seems not to be an evaluation, but rather an exploration of the “experience with the mobilization process”. It is also concerning the quantitative reporting of a qualitative study.*
      - Thank you for this useful comment. We appreciate that it was unclear in the original draft on the type of evaluation we conducted. We agree with your interpretation that it is indeed an exploratory study of the experience with the mobilization training and process. As such, we have clarified this in several instances:
        - Title: On lines **1-4** we edited the title to clarify that this is an exploratory study.
        - Abstract: On line **24** of the background section we state that “*We describe a qualitative exploration*.”
        - Introduction: Stated on lines **101-105** that we qualitatively explore the experience with the mobilization training programme and the related mobilisation processes.
        - Methods: Clarified on lines **205-208** that the report is a qualitative exploration of the mobilization training programme the related mobilization processes, using focus group discussions to explore the perspectives of mobilisers and in-depth interviews for clients newly diagnosed with HIV.
        - Discussion: On Line **373** we have restated that this is an exploratory study.
   b. *The second concern is about the reporting from participants. It is unclear if —for example— what they value is the specific training or the interaction with the research team. That is, was the training what increased self-confidence? Are there specific topics on this soft-skills? From the description of the training this is not clear.*
      - Thank you for flagging this relevant concern. Regarding whether what mobilisers value is the specific training or interaction with the research team, we endeavoured in the manuscript to demonstrate that it was both the training and interaction with the research team that they valued, as well as the interaction with their fellow mobilisers.
      - For example, on lines **244-246**, we have clarified that mobilisers valued both the training and the interaction with the research team: “*They* *highlighted* ***that both*** *the interactive format of the training sessions and regular interactions with the study team during weekly meetings as were key to helping them understand and remember the training content.*”
      - We further emphasize on lines **248-250** the importance of interacting with the research team and the interaction of mobilisers with each other, adding the text in bold to the following: “*They further commented that the weekly meetings with* ***their fellow mobilisers*** *and* ***the study team*** *motivated them in their role by providing regular opportunities to discuss challenges and identify solutions together*”
      - More broadly, the participants reported that the training benefited their mobilisation, as is described in the results on Mobilisation skills from lines **257-292**.
      - Regarding whether there were specific topics on soft skills, we have clarified on line **161-166** that peer mobilization skills also refer to soft skills, including clarity of communication and empathy, and that they were trained through iterative review and discussion with the study team and fellow mobilisers to explore what these concepts mean, why they are important, and how they can be applied. This included mobilisers sharing reflections on how they applied these skills during mobilisation and any challenges that arose and potential solutions.
      - It is also already mentioned in lines **190-191** that these skills were practiced via role plays during the training sessions. We added additional detail from lines **191-195** describing that these skills were practiced via role plays during the training sessions. We detail that “*The* *role plays were led by one of the local study team members in Kiswahili with individuals mobilisers where they would simulate an interaction with a peer. The role plays focused on how to clearly and accurately communicate about OST, AHI testing, and sexual health and how to do so in a patient and empathetic way in order to more effectively addresses the questions and concerns of peers*.”
2. *The discussion needs to focus on the findings, that is, avoid generalizations. As an exploratory study, it is no accident that demonstration of the approach, but rather identifies factors that could help to strengthen the training, as well as highlight the relevance of the context that may limit the potential of the intervention.*

- Thank you for this considerate feedback. We acknowledge the importance of avoiding generalizations, particularly in an exploratory study. We have reviewed the Discussion and revised several statements. These revisions can be found on lines **373, 377-379**, **385, 392, 395, 401, 425, 430, 432**, and in the conclusion from lines **447-454**.

**Review #1 comments**

*I recommend minor revisions in the following aspects:*

1. *Please provide a clear explanation on how the representative sample was arrived at both for the parent study and this one. There's lack of clarity on the actual sample involved in this study and justification of the choice. An average reader will be very confused on the study sample.*
   - Thank you for this constructive comment. Regarding selection of the peer mobilizers, we detail in paragraph one of the Methods section (lines **114-115**) that mobilizers were purposively selected by clinic staff due to participation in previous studies and being representative of their GBMSM and TGW peers. We did not however assess whether the study sample of the parent study was representative of the wider population of GBMSM and TGW, in part due to detailed demographic information of the wider GBMSM and TGW community not being available at the time of the study.
   - Regarding the sample of the clients in the parent study, we detail the eligibility of peers in the methods (lines **128-132**). Eligibility was based on age and a set of behaviours previously identified in a risk score developed by our study team. We have provided additional detail on lines **132-134** articulating that the risk score details statistically significant predictors of incident HIV-1 infection among GBMSM and TGW in coastal Kenya and thus may help local health care providers assess HIV-1 acquisition risk. We have included detailed eligibility criteria in ‘S1 Table’ and referenced this on lines **134-135**. Beyond age, no other demographics (e.g., race, ethnicity, religion, or socioeconomic status) informed recruitment, nor were clients required to be local residents, with all clients identified via the mobilisers peer networks.
   - We also clarify from lines **210-212** that “*All mobiliser were invited to participate in the focus group discussions and all newly diagnosed peers were invited to participated in in-depth interviews that only those willing to participate were included in the sample*.” We have also noted from lines **239-241** that 18 newly diagnosed clients participated in in-depth interviews in the parent study, three of whom were not mobilised.
2. *Was paying the mobilisers and the clients a stipend not a way of inducement? How did the study protocol guard against possible connivance between mobilisers and clients who could pretend as gay or transgender in order to receive the financial incentive?*
   - Thank you for this important comment. While we understand a stipend may risk inducement or connivance, the study team followed local guidelines in line with the standard rates established by the research institute so as to not leave the participants financially worse off as a consequence for taking part in the study, which may impact recruitment and retention based on socioeconomic status. This justification has been added to the text on lines **150-152**. Moreover, as conservative attitudes towards LGTBQIA+ people prevail in coastal Kenya, the study team’s experience suggested that the risk of connivance of clients pretending to be GBMSM or transgender to receive the stipend to be low. Further, clients were part of mobilisers’ networks, so the mobilisers generally had already made an assessment of whether the client would be gay or transgender before introducing them to the study.
3. *In figure 1, in the chart for partner notification services under the "disclosure" section, there is an apparent contradictory message – "You don't need to disclose your HIV status". Please clarify.*
   - Thank you for this comment and for flagging a necessary clarification. Figure 1 provides information on the different modules covered in the training materials and does not detail the peer mobilization processes. The item on Peer Notification Services (PNS) details the PNS protocol from the perspective of someone newly diagnosed with HIV. We have clarified in the caption of the figure on lines **184-187** that the item on ‘disclosure’ refers “*to the fact that an individual newly diagnosed with HIV does not need to disclose their HIV status to a partner as part of the PNS process,* *for example, if they would choose the partner to be notified by a health provider*.”

**Review #2 comments**

*I believe the topic of OST is very relevant in the field of HIV given that is relatively new in most parts of the world. I think the paper reflects very well the implementation of the program and its evaluation.*

1. *While I understand the relevance of including AHI as an important element, I believe there were probably other broader issues that could have been more relevant, such as the importance of testing overall. Can you maybe clarify why the team chose AHI?*
   - Thank you for this clarifying comment, we appreciate the importance of further clarifying why AHI testing was included. We provided in the introduction from lines **62-69** additional context on why testing for AHI is important as part of a comprehensive set of HIV testing services. This included some restructuring of the paragraph to streamline the narrative and providing a reflection from lines **72-79** on why targeted AHI testing may be an efficient strategy in under-resourced settings and the importance of recognizing AHI symptoms at the time of PrEP initiation as PrEP initiation should be delayed if AHI is suspected.
   - We further reflect on why we included AHI testing in the methods section from lines **121-122** stating that AHI testing is “*a relevant component of the promotion of HIV testing throughout the entire life course of HIV infection*.”
2. *Even though the importance of peer mobilization is acknowledged, I think there's room for a stronger statement about how crucial the peers have been in the implementation of new methods for testing, prevention and treatment within the HIV world.*
   - Thank you for this constructive feedback. We discuss the importance of peer mobilization in the introduction and have revised the statement from line **82-85**, including additional references, to emphasize that peer mobilisation represents “*an effective and proven community-led approach to engage people living with and disproportionately affected by HIV*” to underscore the crucial role that peers have played in the successful implementation of new HIV tools.
   - We also include an explicit statement in the opening of the discussion from lines **375-377** that this study “*reflects the* *crucial role that peer mobilisers have been shown to play in the implementation of new methods for testing, prevention, and treatment of HIV*.”
   - We finally included a statement in the conclusion from lines **449-450** that intensive engagement with mobilisers can meaningfully support peer mobilisation and that “*Such an approach can facilitate the crucial role that peer mobilisers have been shown to play in the implementation of new methods for testing, prevention, and treatment of HIV*.”
